# Burden of severe RSV disease among immunocompromised children and adults: a 10 year retrospective study

**DOI:** 10.1186/s12879-018-3002-3

**Published:** 2018-03-06

**Authors:** Olga Chatzis, Stephanie Darbre, Jérôme Pasquier, Pascal Meylan, Oriol Manuel, John David Aubert, Maja Beck-Popovic, Stavroula Masouridi-Levrat, Marc Ansari, Laurent Kaiser, Klara M. Posfay-Barbe, Sandra A. Asner

**Affiliations:** 1 Paediatric Infectious Disease Unit, Division of General Paediatrics, University Hospitals of Geneva & Faculty of Medicine, University of Geneva, Geneva, Switzerland; 2 Department of Paediatrics, Paediatric Infectious Disease Unit, University Hospital of Lausanne, Lausanne, Switzerland; 3 Institute of Social and Preventive Medicine (IUMSP), University of Lausanne, Lausanne, Switzerland; 4 Department of Laboratories, Institute of Microbiology, University Hospital and University of Lausanne, Lausanne, Switzerland; 5 Department of Medicine, Infectious Diseases Service, University Hospital of Lausanne, Lausanne, Switzerland; 6 Department of Surgery, Transplantation Center, University Hospital of Lausanne, Lausanne, Switzerland; 7 Unit of Pulmonary transplantation, Pulmonology Service, University Hospital of Lausanne, Lausanne, Switzerland; 8 Department of Pediatrics, Paediatric Hematology and Oncology Unit, University Hospital of Lausanne, Lausanne, Switzerland; 9 Department of Oncology, Hematology Service, University Hospital of Geneva, Geneva, Switzerland; 10 Paediatric Hematology and Oncology Unit, Division of General Pediatrics, University Hospital of Geneva and Faculty of Medicine, University of Geneva, Geneva, Switzerland; 11 Department of Medicine, Infectious Diseases Service, University Hospital of Geneva and Faculty of Medicine, University of Geneva, Geneva, Switzerland; 12 Department of Genetical and Laboratory Medicine, Virology Laboratory, Laboratory Medicine, University Hospital of Geneva, Geneva, Switzerland; 13 Department of Pediatrics, Pediatric Infectious Disease Unit, CHUV, Rue du Bugnon 46, 1011 Lausanne, Switzerland

## Abstract

**Background:**

Respiratory syncytial virus (RSV) is associated with significant mortality rates amongst hematopoietic stem cell transplant (HSCT) recipients, with less known about other immunocompromised patients.

**Methods:**

Ten-year retrospective cohort study of immunocompromised patients presenting with RSV disease documented at University Hospitals of Lausanne and Geneva. Severe RSV-related outcomes referred to RSV documented respiratory conditions requiring hospital admission, presenting as lower respiratory tract infection (LRTI) or pneumonia. We used multivariable logistic regression to assess clinical and laboratory correlates of severe RSV disease.

**Results:**

From 239 RSV-positive immunocompromised in and out-patients 175 were adults and 64 children of whom 111 (47.8%) presented with LRTI, which resulted in a 38% (89/239) admission rate to hospital. While immunocompromised children were more likely to be admitted to hospital compared to adults (75% vs 62.9%, *p* = 0.090), inpatients admitted to the intensive care unit (17/19) or those who died (11/11) were mainly adults. From multivariable analyses, adults with solid tumors (OR 5.2; 95% CI: 1.4–20.9 *P = 0.015*) or those requiring chronic immunosuppressive treatments mainly for rheumatologic conditions (OR 4.1; 95% CI: 1.1–16.0; *P = 0.034*) were significantly more likely to be admitted to hospital compared to hematopoietic stem cell (HSCT) recipients. Bacterial co-infection was significantly and consistently associated with viral LRTI and pneumonia.

**Conclusions:**

From our findings, RSV-related disease results in a significant burden among adults requiring chronic immunosuppressive treatments for rheumatological conditions and those with solid tumors. As such, systematic screening for respiratory viruses, should be extended to other immunocompromised populations than HSCT recipients.

**Electronic supplementary material:**

The online version of this article (10.1186/s12879-018-3002-3) contains supplementary material, which is available to authorized users.

## Background

Respiratory syncytial virus (RSV), a single-stranded virus of the paramyxoviridae family, is the most important viral etiologic agent of lower respiratory tract infection (LRTI) in infants and young children worldwide [1, 2]. Although mortality rates in healthy infants with RSV pneumonia are less than 0.5%, they can reach up to 60% in untreated immunocompromised children or adults. The burden of RSV infections is well described in adult hematopoietic stem cell transplant (HSCT) recipients in whom progression from upper respiratory tract (URTI) to LRTI is frequent (40 to 60% of cases) with mortality rates approaching 80%. Recent evidence also supports immunomodulatory effects of RSV infections among lung transplant recipients, in whom a significant association between the development of chronic lung allograft dysfunction (CLAD) and RSV infections has recently been reported [3–8].

To date, published reports of RSV infections in immunocompromised patients have primarily focused on adult HSCT recipients with much less known in other adult and pediatric immunocompromised settings [3–7].

Given the lack of information on the burden of RSV infections in other immunocompromised adult and pediatric populations than HSCT recipients, it is essential to obtain further information on the risk factors and outcomes of severe RSV disease among immunocompromised patients. This is important to optimize the prevention of severe RSV disease in this patient population.

This primary objective of this study was to describe the burden and characteristics of RSV infections in a broad population of immunocompromised adults and children. Our secondary objective, was to identify the risk factors for severe RSV infections defined as hospital admission, LRTI and pneumonia.

## Methods

### Study population and definitions

From January 1, 2005 to December 31, 2014, we conducted a multi-center observational study of children and adults presenting with an immunodeficiency state who presented with an RSV-URTI or LRTI in an ambulatory setting or upon or during their admission to the University hospitals of Lausanne and Geneva, Switzerland. RSV-positive patients were identified from Laboratory records. Clinical and laboratory data on RSV-positive immunocompromised adults and children were collected retrospectively with the assistance of hospital medical records. All children less than 18 years of age and adults presenting with at least one of the following conditions were included: allogeneic or autologous HSCT recipients, solid organ transplant (SOT) recipients, patients on cancer chemotherapy or long-term immunosuppression for any chronic disease, which included mostly patients with an underlying rheumatologic condition and those with Connective Tissue Disease (CTD) (Additional file 1: Table S1); and primary immunodeficiency disease (PID). Immunocompromised children with other comorbidities qualifying for RSV prophylaxis based on current Swiss guidelines [8] and patients with human immunodeficiency virus (HIV) were excluded. We defined URTI as detection of RSV in upper respiratory secretions together with symptoms only involving the upper respiratory tract and LRTI as any RSV-positive patient associated with cough, tachypnea and any respiratory distress or wheezing Pneumonia was defined as any of the above LRTI symptoms with pulmonary infiltrates reported by a radiologist on chest radiography and the exclusion of other causes as defined elsewhere [9, 10]. Acute-respiratory-tract infection (ARTI)-attributable hospital admission included patient admitted for respiratory symptoms with concomitant documentation of RSV in a respiratory samples. Severe neutropenia was defined as an absolute neutrophil count (ANC) < 500 cells/μl and severe lymphopenia as an absolute lymphocyte count (ALC) < 100 cells/μl [11]. Approval was obtained from the Research Ethics Boards at University hospitals of Lausanne and Geneva.

### Collection of clinical information

Information on patient demographic variables such as age, gender, type of immunosuppression, bacterial and viral co-infection, median ANC or ALC counts, oral ribavirin or palivizumab treatments were all abstracted from health records. Primary outcome referred to acute respiratory tract infection (ARTI)-attributable hospital admission. Secondary outcomes consisted of the documentation of LRTI or pneumonia, intensive care unit (ICU) admission and RSV-contributed mortality within 30 days of the diagnosis of RSV infection. RSV-attributable admission to the intensive care unit (ICU) was defined as a documented RSV disease requiring admission to the ICU. Overall mortality referred to death from any cause whereas RSV-contributed mortality was defined as a persistent or progressive RSV infection with respiratory failure documented at the time of death [12]. Each case was adjudicated by 2 members of the study team.

### Virology studies

From 2005 to 2013, nasopharyngeal specimens, broncho-alveolar lavage (BAL) fluids and sputum samples were examined by a two-step reverse transcriptase polymerase chain reaction (RT-PCR) assay, targeting RSV A and B. This assay is extensively validated at the Laboratory of Virology, University Hospitals Geneva. In addition, specimens collected from children, which required immediate identification for cohorting purposes, were tested by a rapid antigen assay for RSV (RSV Respi-Strip, Coris Bioconcept) [13, 14] A commercial one-step PCR FTD Respiratory pathogens 21 (ref.FTD.-53–96/12) from Fast-Track Diagnostics (Luxembourg) (used at University hospitals Geneva since 2013) and the Cepheid Xpert Flu/RSV (used at University hospital Lausanne since 2015) further replaced molecular and rapid antigen assays [15, 16]. A patient was defined as being RSV-positive if any specimen was positive by any diagnostic method (antigen assay or any of the above listed molecular assay). Multiple simultaneous virus infections (herein referred to as viral co-infection) were those in which two or more virus pathogens were detected from the same respiratory sample. Bacterial co-infection was defined as the presence of any bacterial pathogen, identified by culture from blood or respiratory samples upon initial consultation with respiratory symptoms or within 30 days of their initial consultation in association with a documented viral infection.We excluded children if blood culture isolates were considered to be contaminants based on international guidelines [17].

### Statistical analyses

Standard descriptive and comparative statistics (the Fisher’s exact test for categorical variables and the Mann-Whitney U test for continuous variables) were used on data categorized by age groups (children under 18 years of age and adults) and in and outpatients. For all statistical testing, only the first RSV-positive nasopharyngeal swab was included in cases with multiple RSV-positive swabs within the same patient because these samples cannot be considered independent. Univariable and multivariable logistic regression were used to assess clinical and laboratory correlates of ARTI-attributable hospital admission, LRTI and pneumonia with stratified analyses for children and adults. Predictor variables included different immunosuppressive categories, age, bacterial infection, and ALC for all patients and the adult subgroup whereas only bacterial co-infection and ALC were included in the children subgroup given the small number of observations. Statistically significant predictors in univariable analyses were included in multivariable analyses [18]. All analyses were conducted by complete case analyses, excluding all patients with missing values for any dependent or independent variables. We evaluated next the robustness of the estimates related to the potential differences in disease severity described for i) HSCT recipients infected with RSV within 2 years of transplantation; ii) patients who presented with viral co-infections and iii) patients detected RSV-positive by molecular assays solely. Therefore, we performed three post hoc sensitivity analyses for primary and secondary outcomes: one in which HSCT recipients infected with RSV after two years from their transplantation were excluded, another one excluding all 52 patients with viral co-infections and one excluding 12 children detected RSV-positive by rapid antigen assays only. Two sided tests were performed, and a *P* value *< 0.05* was considered to be statistically significant. Data were analyzed using SPSS statistical software (version 20.0, SPSS Inc., Chicago, IL, USA), SAS and R (version 3.3.3, R Foundation for Statistical Computing, Vienna, Austria).

## Results

### Descriptive characteristics of study subjects

Three thousand two hundred and twenty-three RSV-positive patients (4′605 positive RSV samples) of whom 239 (7.4%) were eligible RSV-positive immunocompromised subjects (64 (26.8%) children and 175 adults (73.2%) (Additional file 1: Figure S1). From all patients, HSCT recipients (*N* = 67; 28.0%) and SOT recipients (*N* = 63; 26.4%) were the most common immunosuppressive conditions included. Most of the 64 children, 35 (54.7%) males, with a median age of 4.9 years (interquartile range [IQR] 3.2–7.7) presented with leukaemia or lymphoma (*N* = 29; 45.3%), whereas HSCT (*N* = 59, 33.7%) and SOT (*N* = 58, 33.1%) were the most common immunosuppressive conditions documented among the 175 adults (Table 1).

**Table 1:** Baseline characteristics of immunocompromised patients with RSV infection

Variable; number (%) unless indicated otherwise	CHILDREN *N* = 64		ADULTS *N* = 175		TOTAL *N* = 239	
Variable; number (%) unless indicated otherwise	INPATIENTS	OUTPATIENTS	INPATIENTS	OUTPATIENTS	INPATIENTS	OUTPATIENTS
	*N* = 48	*N* = 16	*N* = 107	*N* = 68	*N* = 155	*N* = 84
< 2 years	11 (23)	2 (12.5)				
2–5 years	13 (27.1)	7 (43.75)				
5–18 years	24 (50)	7 (43.75)				
Age (years) median (IQR)	4.9 (2.7–7.4)	47 (.3.7–8.8)	60.5 (48–70.6)	50.8 (37.3–59.4)		
Male	26 (54.2)	9 (56.3)	53 (49.5)	34 (20)	79 (51)	43 (51,2)
HSCT	2 (4.2)	6 (37.5)	24 (22.4)	35 (51.5)	26 (16.8)	41 (48.8)
Autologous	0	0	9 (8.4)	1 (1.5)	9 (5.8)	1(1.2)
Allogeneic	0	8(100)	15 (14)	34 (50)	12(7,7)	45(53,6)
Matched	0	3 (18.8)	5 (4.7)	19(28)	5(3,2)	22(26,2)
Mismatched	0	0	1 (1)	0	1(0,6)	0
Unrelated	2 (4.2)	1 (6.3)	9 (8.4)	15(22,1)	11(7,1)	16(19)
SOT	3(6.3)	2 (12.5)	32 (30)	26(38,2)	35(22,6)	28(33,3)
Leukaemia /lymphoma	23 (47.9)	6 (37.5)	18 (16.8)	3(4,4)	41(26,5)	9(10,7)
Solid tumor	9 (18.8)	2 (12.5)	15 (14)	1(1,5)	24(15,5)	3(3,6)
Chronic immuno-suppression	6 (12.5)	0	18 (16.8)	2 (3.0)	24(15,5)	2(2,4)
PID	5 (10.4)	0	0	1(1)	5(3,2)	1(1,2)
GVHD among allogeneic HSCT recipients	1 (2.1)	4 (25)	9 (8.4)	21 (30.9)	10(6,5)	25(29,8)
Bacterial co-infection	8 (16.7)	1(6.3)	25 (23.4)	2 (3)	33(21,3)	3(3,6)
Viral co-infection	12 (25)	6 (37.5)	22 (20.6)	12 (17.7)	34(21,9)	18(21,4)
ANC, median, (IQR)	1.1 (0.5–3.1)	1.7 (1.6–4.2)	4.6 (1.4–8.1)	3.5 (2.6–5.3)	3.4 (0.9–6.7)	3.3 (2.3–5.3)
ALC, median (IQR)	0.8 (0.2–2.1)	1.6 (0.7–1.9)	0.7 (0.1–1.1)	1.1 (0.6–1.9)	0,7(0,2–1,4)	1.1 (0.6–1.9)
Oral ribavirin treatment	1 (2.1)	1 (6.3)	24 (22.4)	12 (17,6)	25 (16.1)	13(15,5)
Palivizumab treatment	2 (4.2)	0	7 (6.5)	1 (1.5)	9 (5,8)	1(1,2)

Absolute neutrophil counts (ANC), Absolute lymphocyte counts (ALC), Graft vs. host disease (GVHD), Hematopoietic stem cell transplant (HSCT), Interquartile range (IQR), Primary Immunodeficiency Disease (PID), Solid Organ Transplant (SOT)

Number of missing values: Children outpatients: ANC *N* = 1, ANC =1; adult inpatients ANC *N* = 5, ALC *N* = 5; adult outpatients ANC *N* = 13, ALC *N* = 13

### Proportion of RSV-positive specimens, bacterial and viral-co-infections

RSV was detected positive from 202 (84.5%) nasopharyngeal (NP) swabs and 35 (15%) BAL, of which 25 (28.6%) were collected from patients with pneumonia. Two hundred and twenty-seven patients (95%) were detected by molecular assays and 12 (5%) by rapid antigen assays. From all patients, 36 (15.1%) presented with a documented bacterial co-infection, of whom 29 (80.6%) with bacteremia and 7 (19.4%) with bacterial pneumonia documented from BAL. From the 11 patients who died, only 3 (27.3%) presented with a bacterial co-infection (*S. aureus, Stenotrophomonas maltophilia and Legionella pneumophila*) documented from BAL. 52 (21.8%) patients presented with a viral co-infection of whom 10 (19.2%) had more than two viruses detected. The most common viral co- infections were human rhinovirus/enterovirus-RSV (16/42; 38.1%), parainfluenza (PIV)-RSV (6/42; 14.3%) and influenza (FLU)-RSV (6/42; 14.3%) (Table 1).

### Clinical outcomes

From all immunocompromised subjects, 89 (37.2%) (*N* = 58; 34.1% adults; *N* = 31; 48.4% children; *p* = 0.090) were admitted to hospital for their RSV infection with a median duration of stay of 5 days (IQR: 0–18 days). From those 89, 19 (21.3%) patients were admitted to intensive care of whom 6 (31.6%) presented with chronic disease requiring underlying immunosuppressive therapy, 4 (21.1%) with solid tumors and 4 (21.1%) with HSCT. Patients admitted to the ICU, requiring mechanical ventilation and those who died were almost all adults. Likewise immunocompromised adults presented with significantly higher rates of pneumonia (36.9% vs. 18.8%, *p* = 0.008) compared to immunocompromised children. Furthermore, all 11 adults who died within 30 days of their RSV-attributable admission, presented with pneumonia despite ribavarin (*N* = 6, 54.5%) or palivizumab (*N* = 2,18.2%) administration. Most of those 11 patients, median age of 64.6 years (IQR 34.1–73.9), were HSCT recipients (*N* = 4, 36.4%) or presented with lymphoma/leukemia (*N* = 3, 27.3%) or solid tumors (*N* = 3; 27.3%) (Table 2).

**Table 2:** Clinical outcomes of immunocompromised patients with RSV infection

Variable, Number; (%) unless indicated otherwise	Children (*N* = 64)	Adult (*N* = 175)	*p* value	Total (*N* = 239)
All-cause admission to hospital (adults: 5 missing values)	48 (75.0)	107 (62.9)	0.090	155 (66.2)
ARTI-attributable hospital admission^a^ (adults: 5 missing values)	31 (48.4)	58 (34.1)	0.050	89 (38.0)
• Length of hospital stay (d) mean (SD)^a^	• 5 (3.5)	• 9 (12.0)	• < 0.001	• 7 (9.0)
• Admission to the ICU^a^	• 2 (6.5)	• 17 (29.3)	• 0.014	• 19 (21.3)
• Use of mechanical ventilation^a^	• 1 (3.2)	• 13 (22.4)	• 0.029	• 14 (15.7)
• Mortality within 30 days of admission^a^	• 0	• 11 (19.0)	• 0.007	• 11 (12.4)
All-cause mortality within 30 days of admission	1 (1.6)	20 (11.4)	0.018	21 (8.8)
LRTI ^b^(adults: 7 missing values)	26 (40.6)	85 (50.6)	0.188	111 (47.8)
RSV-attributable pneumonia^b^	12 (18.8)	62 (36.9)	0.008	74 (31.9)
• RSV documented from BAL^b^	2 (16.7)	23 (37.1)	0.024	25 (33.8)
• RSV-bacterial co-infection documented from BAL	0	7 (11.3)	0.020	7 (9.5)

^a^Refers to patients admitted to hospital for an acute-respiratory-tract infection (ARTI)-attributable hospital admission (i.e excluded patients admitted to hospital for other reasons). ^b^Refers to all patients seen in an ambulatory setting or admitted to hospital for their RSV infection or for another reason. BAL: Bonchoalveolar lavage, SD: Standard deviation, LRTI: Lower respiratory tract infection (adults 7 missing values); d: days. RSV-attributable pneumonia (7 missing values)

### Predictors of hospitalization

From multivariable analyses, we identified that patients with leukaemia/lymphoma (odds ratio [OR] 3.9; 95% confidence interval [CI]: 1.7–9.7; *P = 0.002*), solid tumors (OR 7.4; 95% CI: 2.7–21.9; *P < 0.001*) or those requiring chronic immunosuppression mainly for vasculitis (OR 9.5; 95% CI: 3.3–30.2 *P < 0.001*) when compared to HSCT, were significantly more likely to be admitted to hospital for RSV. When stratified by age groups (adults vs. children), increasing age (OR 1.4; 95% CI: 1.0–1.8 *P = 0.027*), patients with solid tumors (OR 5.2; 95% CI: 1.4–20.9; *P = 0.015*) and those requiring any chronic immunosuppression mainly for vasculitis (OR 4.1; 95% CI: 1.1–16.0; *P = 0.034*) when compared to HSCT, remained significant predictors of ARTI-attributable hospital admission among adults. *Post-hoc s*ensitivity analyses did not affect our risk estimates and the above findings (Table 3, Additional file 1: Tables S2, S4 and S6).

**Table 3:** Predictors for ARTI-attributable hospital admission among all immunocompromised patients and stratified by age-groups (adults vs children)

*N* = 215 (152 adults, 63 children)^a^		Univariable analyses	Multivariable analyses	
		OR (95% CI)	OR (95% CI)	*P* value
Increasing age (by 10Y range)	All	1.0 (0.9;1.1)	1.0 (0.9;1.2)	0.558
	Adults	1.6 (1.3;2.1)	1.4 (1.0;1.8)	0.027
	Children	0.6 (0.2;1.8)	–	–
Bacterial co-infection	All	1.6 (0.8;3.4)	1.5 (0.7;3.3)	0.340
	Adults	2.2 (0.9;5.2)	1.7 (0.6;4.5)	0.286
	Children	0.8 (0.2;3.3)	1.0 (0.2;4.6)	0.995
ALC	All	1.0 (1.0;1.2)	1.0 (1.0;1.2)	0.420
	Adults	1.0 (1.0;1.2)	1.0 (1.0;1.2)	0.436
	Children	0.8 (0.5;1.2)	0.8 (0.5;1.2)	0.309
SOT	All	2.1 (0.9;5.1)	1.9 (0.8;4.6)	0.166
	Adults	2.1 (0.9;5.4)	1.6 (0.6;4.4)	0.312
	Children	1.7 (0.1;53.3)	–	–
Leukemia/lymphoma	All	4.0 (1.7;9.5)	3.9 (1.7;9.7)	0.002
	Adults	3.0 (1.0;9.3)	1.5 (0.4;5.2)	0.556
	Children	7.5(1.1;149.7)	–	–
Solid tumor	All	7.2 (2.6;21.2)	7.4 (2.7;21.9)	< 0.001
	Adults	7.4 (2.1;28.4)	5.2 (1.4;20.9)	0.015
	Children	10.5 (1.2;240.1)	–	–
Chronic immunosuppressive medication	All	10.5 (3.7;32.9)	9.5 (3.3;30.2)	< 0.001
	Adults	8.2 (2.6;28.4)	4.1 (1.1;16.0)	0.034
	Children	35.0 (2.6;1450.5)	–	–
PID	All	4.3 (0.7–26.1)	4.5 (0.7;29.0)	0.097
	Children	10.5 (0.8–298.2)	–	–

Absolute lymphocyte counts (ALC), Hematopoietic stem cell recipients (HSCT), Primary immunodeficiency disease (PID) Solid organ transplant (SOT), Acute-respiratory-tract infection (ARTI)

^a^N refers to the total number of patients included in all multivariable analyses for whom information on all predictors and outcomes were available. 24 patients (23 adults – 1 child) were excluded from univariable and multivariable analyses because of missing values for nALC

Limited sample size in the pediatric subset limited the number of predictors, which could be included in multivariable analyses (−)

### Predictors of LRTI and pneumonia

From multivariable analyses, patients presenting with a bacterial co-infection were significantly more likely to present with LRTI (OR 3.4; 95% CI: 1.5–8.2; *P = 0.005*) and pneumonia (OR 3.6; 95% CI: 1.6–8.4; *P = 0.002*). When stratified by age groups (adults vs. children), bacterial co-infection was also an independent and significant predictor for LRTI (OR 3.4; 95% CI: 1.3–9.8; *P = 0.018*) only in adults. Increasing age was also a significant predictor for LRTI (OR 1.1; 95% CI: 1.0–1.3; *P = 0.048*) and pneumonia (OR 1.2; 95% CI: 1.1–1.4 *P = 0.003*) among all patients. *Post-hoc s*ensitivity analyses did not affect our risk estimates and the above findings (Table 4, Additional file 1: Tables S3, S5 and S7).

**Table 4:** Predictors of progression to LRTI and pneumonia among all immunocompromised patients and stratified by age-groups (adults vs children)

		Univariable analyses	Multivariable analyses	
		OR (95%CI)	OR (95% CI)	*P* value
Outcome: LRTI *N* = 215 (152 adults, 63 children)^a^				
Increasing age (by 10Y range)	All	1.2 (1.0;1.3)	1.1 (1.0;1.3)	0.048
	Adults	1.3 (1.1;1.7)	1.2 (1.0;1.5)	0.128
	Children	0.9 (0.3;2.8)	–	–
Bacterial co-infection	All	3.3 (1.5;7.5)	3.4 (1.5;8.2)	0.005
	Adults	3.2 (1.3;8.6)	3.4 (1.3;9.8)	0.018
	Children	3.4 (0.8;17.5)	4.1 (0.9;24.6)	0.084
ALC	All	1.0 (1.0;1.2)	1.0 (1.0;1.2)	0.501
	Adults	1.1 (1.0;1.3)	1.0 (1.0;1.3)	0.580
	Children	1.0 (0.6;1.4)	0.9 (0.6;1.3)	0.457
Type immunosuppression (compared to HSCT)				
SOT	All	0.9 (0.4;1.9)	0.7 (0.3;1.4)	0.294
	Adults	1.0 (0.4;2.1)	0.7 (0.3;1.5)	0.344
	Children	0.7 (0.1;6.4)	–	–
Leukemia/lymphoma	All	1.0 (0.5;2.0)	1.0 (0.5;2.3)	0.935
	Adults	2.0 (0.7;6.0)	1.2 (0.4;3.9)	0.791
	Children	0.5 (0.1;2.7)	–	–
Solid tumor	All	1.0 (0.4;2.5)	1.0 (0.4;2.6)	0.985
	Adults	1.2 (0.4;3.8)	0.8 (0.2;2.8)	0.718
	Children	0.7 (0.1;4.4)	–	–
Chronic immunosuppressive medication	All	3.4 (1.2;10.4)	2.6 (0.9;8.3)	0.077
	Adults	4.0 (1.3;15.6)	2.4 (0.7;10.2)	0.204
	Children	2.0 (0.2;21.4)	–	–
PID	All	0.6 (0.1;3.1)	0.7 (0.1;4.3)	0.724
	Children	0.7 (0.1;6.4)	–	–
Outcome: LRTI *N* = 215 (152 adults, 63 children)^a^				
Increasing age (by 10Y range)	All	1.2 (1.1;1.4)	1.2 (1.1;1.4)	0.003
Bacterial co-infection	All	3.1 (1.5;6.6)	3.6 (1.6;8.4)	0.002
ALC	All	1.0 (1.0;1.1)	1.0 (1.0;1.1)	0.439
Type immunosuppression (compared to HSCT)				
SOT	All	0.7 (0.3;1.7)	0.5 (0.2;1.1)	0.094
Leukemia/lymphoma	All	0.9 (0.4;2.1)	1.0 (0.4;2.5)	0.956
Solid tumor	All	1.2 (0.5;3.2)	1.3 (0.4;3.6)	0.639
Chronic immunosuppressive medication	All	2.0 (0.8;5.4)	1.4 (0.5;3.8)	0.540
PID	All	0.4 (0.0;2.8)	0.7 (0.0;5.0)	0.721

Absolute lymphocyte counts (ALC); Hematopoietic stem cell recipients (HSCT), Primary immunodeficiency disease (PID); Solid organ transplant (SOT);

^a^N refers to the total number of patients included in all multivariable analyses for whom information on all predictors and outcomes was available. 24 patients (23 adults – 1 child) were excluded from univariable and multivariable analyses because of missing values for ALC

Limited sample size in the pediatric subset limited the number of predictors which could be included in multivariable analyses for the outcome LRTI (−)

## Discussion

Two important observations made in our study were that 1) RSV-infected immunocompromised adults (mainly elderly) presented with more severe outcomes compared to children; and that 2) adult patients with solid tumors or leukaemia and those requiring chronic immunosuppression for vasculitis were significantly more likely to be admitted to hospital for RSV disease compared to HSCT recipients.

The overall burden of RSV among health-seeking populations has mostly been evidenced in young children, adults above 65 years of age, pregnant women or immunocompromised patients in whom equivalent disease burden to influenza has been reported [19–21]. The estimation of severe RSV infections in Switzerland is limited by the lack of an active RSV surveillance system. However, from our findings, we estimated that less than 10% RSV positive samples were collected from immunocompromised subjects, thus suggesting either that RSV only affects a very small subset of immunococompromised patients or insufficient diagnostic screening [6, 11, 22, 23].

We have documented important differences in outcomes of RSV infections among immunocompromised children compared with immunocompomised adults. While children were more frequently admitted to hospital for their RSV infection compared to adults, most inpatients admitted to the ICU, requiring invasive ventilation or those who died were adults, thus suggesting worse outcomes among our adult inpatients. In addition, elderly patients were significantly more likely to present with pneumonia compared to children. Furthermore, as supported in another study [6], over one third of our RSV-infected immunocompromised adult cohort presented with pneumonia for whom a transfer to the ICU was required resulting in mortality rates of almost 20%. These important findings highlight the potential consequences of RSV infection among adults with underlying immunodeficiencies and may suggest that these patients would specifically benefit from preventive strategies.

As suggested in another study [6], bacterial co-infection also contributed undoubtedly to progression towards LRTI and pneumonia, likely as a result of RSV-induced injuries to respiratory epithelium thereby increasing bacterial adherence [11, 24, 25].

Studies focusing on the severity of viral co-infections have resulted in different findings likely as a result of including various age groups and breadth of illness severity and limited sample sizes [22, 26, 27]. While viral co-infections were documented among one-third of our pediatric patient-population and 20% of the adult one, no significant association between viral co-infections and our outcomes of interest were documented, in line with findings from a recent systematic review [28]. Furthermore, *post-hoc* sensitivity analyses excluding patients with viral co-infections did not affect our risk estimates.

The burden of RSV infections has been widely and consistently evidenced among HSCT recipients, with much less known in other immunocompromised patient populations [6, 9, 11]. Some studies [29, 30] reported severe RSV outcomes among SOT recipients and children with congenital immunodeficiencies, without being consistent throughout the literature [31]. To our knowledge, this is the first report showing that adult patients requiring chronic immunosuppression mainly for Connective Tissue Disease (CTD) or vasculitis and those with solid tumors were at highest risk for hospital admission when compared to HSCT recipients. Furthermore, discrepancies between our findings and those in published literature were not influenced by timing after transplantation as *post-hoc* sensitivity analyses excluding HSCT recipients infected 2 years after their transplantation, did not affect our risk estimates. We also reported increased severity of RSV infections among patients with leukaemia in line with published literature [32, 33]. These important findings highlight the burden of RSV disease among various categories of immunocompromised patients other than HSCT recipients.

Important strengths of our study include a large variety of immunocompromised adults and children categorized in 6 mutually exclusive categories. All subgroup analyses were defined a priori and post hoc sensitivity analyses confirmed robustness of the results. The inclusion of outpatients enabled the use of hospital admission as a measure of clinical severity in addition to LRTI and pneumonia thus supporting consistency of our findings. Potential limitations of our study relate to its retrospective design and laboratory-based collected data, which may have led to selection bias. However all consecutive immunocompromised patients with URT or LRT symptoms were systematically tested for respiratory viruses according to local guidelines, which may have reduced the risk of selection bias. Second, the inclusion of adults and children and the lack of adjustment for other comorbidities, may have biased our findings towards more severe outcomes among adults, as children are routinely screened for RSV infections whereas adults are swabbed for RSV while experiencing more severe symptoms. However, half of our adult RSV-positive cohort was screened in an ambulatory setting while consulting with mild symptoms and sensitivity analyses excluding children detected by rapid antigen assays did not affect our risk estimates. As such, we believe, that our findings were still suggestive of worse clinical outcomes in RSV-infected immunocompromised adults. Third, our estimation of bacterial co-infections may have been underestimated as BAL are rarely performed in children and pneumonia rarely results in positive blood cultures. Finally, the lack of a comparator group of patients without any other identified pathogen would have better delineated the attribution of disease burden solely to RSV.

## Conclusions

In conclusion, our study conducted on a small sub-set of patients, suggests a higher burden of RSV disease in immunocompromised adults compared to children, more specifically among patients with solid tumors, leukaemia/lymphoma or those requiring chronic immunosuppression for CTD or vasculitis.

## Additional file

Additional file 1: **Figure S1.** Flow diagram. **Table S1.** Description of long-term immunosuppression treatment population. **Table S2.** Predictors for ARTI-attributable hospital admission among all immunocompromised patients and stratified by age-groups (adults vs. children). **Table S3.** Predictors of progression to LRTI and pneumonia among all immunocompromised patients and stratified by age-groups (adults vs. children). **Table S4.** Predictors for ARTI-attributable hospital admission among all immunocompromises patients and stratified by age-groups (adults vs. children). **Table S5.** Predictors of progression to LRTI and pneumonia among all immunocompromises patients and stratified by age-groups (adults vs. children). **Table S6.** Predictors for ARTI-attributable hospital admission among all immunocompromised patients and stratified by age-groups (adults vs. children). **Table S7.** Predictors of progression to LRTI and pneumonia among all immunocompromised patients and stratified by age-groups (adults vs. children). (DOCX 140 kb)

## Abbreviations

ALC: Absolute lymphocytic count
ANC: Absolute neutrophil count
ARTI: Acute-respiratory-tract infection
CLADE: Chronic lung allograft dysfunction
CTD: Connective Tissue Disease
HIV: Human immunodeficiency virus
HSCT: Hematopoeitic stem cell transplant
LRTI: Lower respiratory tract infection
PID: Primary immunodeficiency disease
RSV: Respiratory syncytial virus
SOT: Solid organ transplant
URTI: Upper respiratory tract infection
