# Supplementary material for: Burden of severe RSV disease among immunocompromised children and adults: a 10 year retrospective study
Source: BMC Infect Dis. 2018 Mar 6;18:111. doi: 10.1186/s12879-018-3002-3 (PMC5838875; doi:10.1186/s12879-018-3002-3)
Supplement: Supplementary file 1 — Figure S1. Flow diagram. Table S1. Description of long-term immunosuppression treatment population. Table S2. Predictors for ARTI-attributable hospital admission among all immunocompromised patients and stratified by age-groups (adults vs. children). Table S3. Predictors of progression to LRTI and pneumonia among all immunocompromised patients and stratified by age-groups (adults vs. children). Table S4. Predictors for ARTI-attributable hospital admission among all immunocompromises patients and stratified by age-groups (adults vs. children). Table S5. Predictors of progression to LRTI and pneumonia among all immunocompromises patients and stratified by age-groups (adults vs. children). Table S6. Predictors for ARTI-attributable hospital admission among all immunocompromised patients and stratified by age-groups (adults vs. children). Table S7. Predictors of progression to LRTI and pneumonia among all immunocompromised patients and stratified by age-groups (adults vs. children). (DOCX 140 kb) [file 12879_2018_3002_MOESM1_ESM.docx]

SUPPLEMENTARY DATA

**Figure S1: Flow diagram**


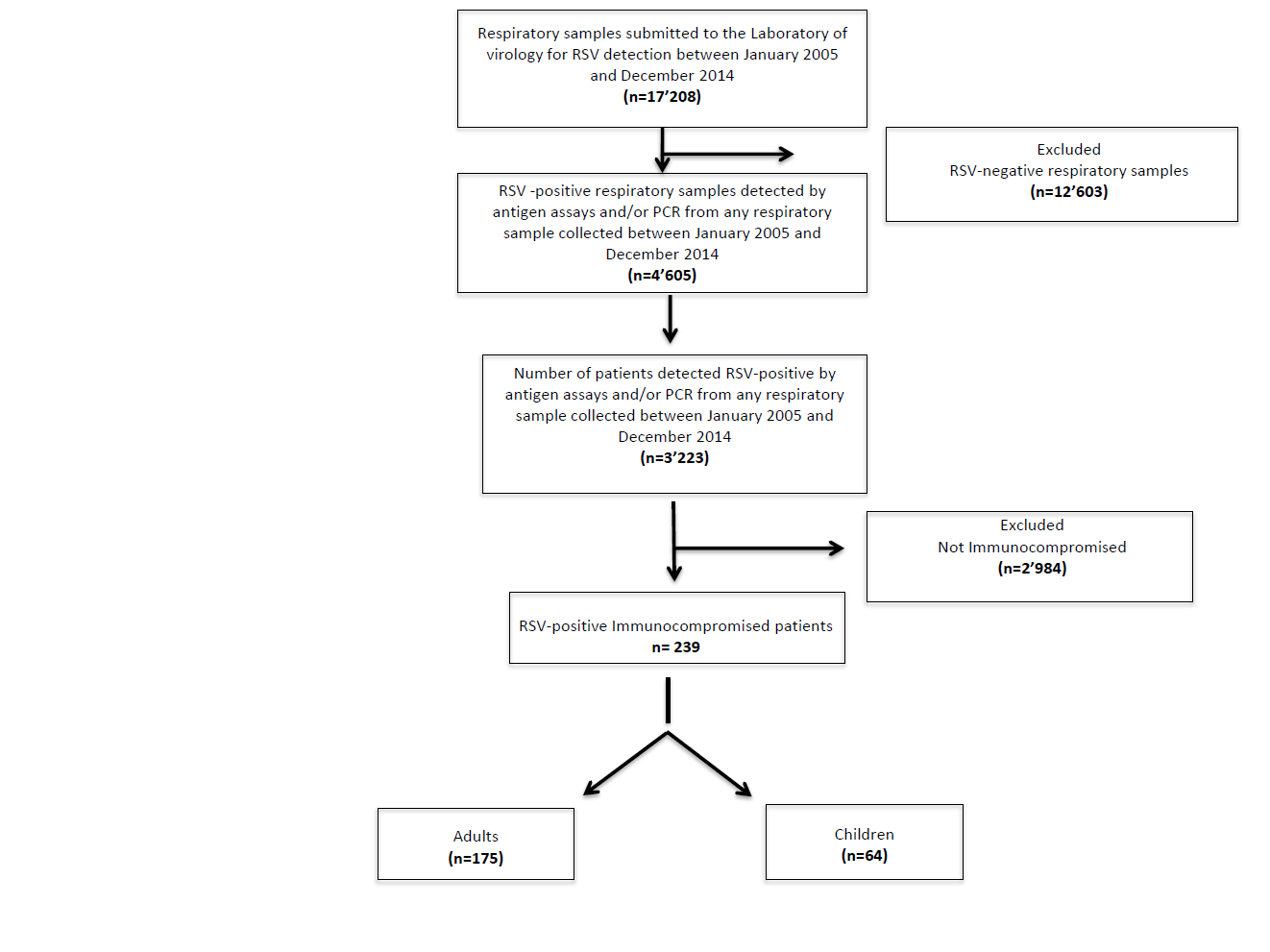


**Addendum Table S1: Description of long-term immunosuppression treatment population**

| Connective Tissue Disease (CTD) | Mixed Connective Tissue Disease (MCTD) (1) |
| --- | --- |
|  | Systemic Lupus Erythematosus (2) |
|  | Systemic-onset juvenile idiopathic arthritis (2) |
|  | Rheumatoid arthritis (4) |
| Vasculitis | Polyarteritis nodosa (PAN) (1) |
|  | Wegener disease (1) |
|  | Horton disease (3) |
|  | Churg-Strauss syndrome (1) |
| Rheumatic disease | Psoriatic arthritis (2) |
|  | Chronic Infantile Neurological Cutaneous and Articular syndrome (CINCA syndrome) (1) |
| Other | Bullous pemphigoid (1) |
|  | Visceral amyloidosis (1) |
|  | Auto-immune hepatitis (1) |
|  | Nephrotic syndrome (1) |
|  | Interstitial pneumonia (3) |

**Addendum Tables S2 and S3 refer to *Post-hoc* sensitivity analyses excluding HSCT recipients infected 2 years after their transplantation.**

**Addendum Table S2**

**Predictors for ARTI-attributable hospital admission among all immunocompromised patients and stratified by age-groups (adults vs. children).**

| **N=206 (143 adults, 63 children)*** | | **Univariate analyses** | | | | **Multivariate analyses** | | | |
| --- | --- | --- | --- | --- | --- | --- | --- | --- | --- |
|  |  | **OR** | **95 %C.I** | **P value** | | **OR** | **95 % C.I** | | **P value** |
| **Increasing age (by 10Y range)** | **All** | 1.0 | 0.9;1.1 | 0.570 | | 1.0 | 0.9;1.2 | | 0.575 |
|  | **Adults** | 1.6 | 1.3;2.1 | <0.001 | | 1.4 | 1.1;1.8 | | 0.025 |
|  | **Children** | 0.6 | 0.2;1.8 | 0.360 | | - | - | | - |
| **Bacterial co-infection** | **All** | 1.6 | 0.8;3.3 | 0.217 | | 1.5 | 0.7;3.4 | | 0.335 |
|  | **Adults** | 2.1 | 0.9;5.0 | 0.081 | | 1.7 | 0.6;4.5 | | 0.286 |
|  | **Children** | 0.8 | 0.2;3.3 | 0.758 | | 1.0 | 0.2;4.6 | | 0.995 |
| **nALC** | **All** | 1.0 | 1.0;1.2 | 0.323 | | 1.0 | 1.0;1.2 | | 0.419 |
|  | **Adults** | 1.0 | 0.5;1.2 | 0.311 | | 1.0 | 1.0;1.3 | | 0.450 |
|  | **Children** | 0.8 | 0.5;1.2 | 0.291 | | 0.8 | 0.5;1.2 | | 0.309 |
| **Type immunosuppression (Reference: HSCT)** | | | | | | | | | |
| **SOT** | **All** | 2.2 | 0.9;5.6 | | 0.092 | 2.0 | | 0.8;5.1 | 0.157 |
|  | **Adults** | 2.2 | 0.9;5.9 | | 0.110 | 1.7 | | 0.6;4.8 | 0.300 |
|  | **Children** | 1.7 | 0.1 ;53.3 | | 0.718 | - | | - | - |
| **Leukaemia/lymphoma** | **All** | 4.1 | 1.7;10.4 | | 0.002 | 4.1 | | 1.7;10.6 | 0.003 |
|  | **Adults** | 3.1 | 1.0;10.0 | | 0.059 | 1.5 | | 0.4;5.5 | 0.552 |
|  | **Children** | 7.5 | 1.1 ;149.7 | | 0.075 | - | | - | - |
| **Solid tumor** | **All** | 7.5 | 2.6;23.0 | | <0.001 | 7.7 | | 2.7;23.7 | <0.001 |
|  | **Adults** | 7.6 | 2.1;30.4 | | 0.002 | 5.4 | | 1.4;22.4 | 0.016 |
|  | **Children** | 10.5 | 1.2 ;240.1 | | 0.060 | - | | - | - |
| **Chronic immunosuppressive medication** | **All** | 10.9 | 3.7;35.5 | | <0.001 | 10.0 | | 3.4;32.7 | <0.001 |
|  | **Adults** | 8.4 | 2.6;30.6 | | <0.001 | 4.2 | | 1.1;16.9 | 0.039 |
|  | **Children** | 35 | 2.6 ;1450.5 | | 0.020 | - | | - | - |
| **PID** | **All** | 4.5 | 0.7;27.7 | | 0.090 | 4.7 | | 0.7;30.6 | 0.093 |
|  | **Children** | 10.5 | 0.8 ;298.2 | | 0.094 | - | | - | - |

**Legend:** Absolute lymphocyte counts (ALC), Hematopoietic stem cell recipients (HSCT), Primary immunodeficiency disease (PID) Solid organ transplant (SOT), acute-respiratory-tract infection (ARTI)**;** N refers to the total number of patients included in all multivariable analyses for whom information on all predictors and outcomes was available

**Addendum Table S3**

**Predictors of progression to LRTI and pneumonia among all immunocompromised patients and stratified by age-groups (adults vs. children)**

| **Outcome: LRTI**  **N= 206 (143 adults, 63 children)*** | | **Univariate analyses** | | | **Multivariate analyses** | | |
| --- | --- | --- | --- | --- | --- | --- | --- |
|  |  | **OR** | **95 %C.I** | **P value** | **OR** | **95 % C.I** | **P value** |
| **Increasing age**  **(by 10Y range)** | **All** | 1.2 | 1.0;1.3 | 0.009 | 1.1 | 1.0;1.3 | 0.053 |
|  | **Adults** | 1.4 | 1.1;1.8 | 0.002 | 1.2 | 1.0;1.6 | 0.097 |
|  | **Children** | 0.9 | 0.3;2.8 | 0.914 | - | - | - |
| **Bacterial co-infection** | **All** | 3.5 | 1.7;8.1 | 0.002 | 3.5 | 1.6;8.5 | 0.003 |
|  | **Adults** | 3.5 | 1.4;9.6 | 0.008 | 3.5 | 1.3;10.3 | 0.014 |
|  | **Children** | 3.4 | 0.8;17.5 | 0.108 | 4.1 | 0.9;24.6 | 0.084 |
| **nALC** | **All** | 1.0 | 1.0;1.2 | 0.359 | 1.0 | 1.0;1.2 | 0.510 |
|  | **Adults** | 1.1 | 1.0;1.3 | 0.393 | 1.0 | 1.0;1.4 | 0.596 |
|  | **Children** | 1.0 | 0.6;1.4 | 0.830 | 0.9 | 0.6;1.3 | 0.457 |
| **Type immunosuppression (compared to HSCT)** | | | | | | | |
| **SOT** | **All** | 1.1 | 0.5;2.5 | 0.752 | 0.8 | 0.4;1.8 | 0.613 |
|  | **Adults** | 1.2 | 0.5;2.8 | 0.628 | 0.8 | 0.3;2.1 | 0720 |
|  | **Children** | 0.7 | 0.1;6.4 | 0.725 | - | - | - |
| **Leukaemia/lymphoma** | **All** | 1.2 | 0.5 ;2.6 | 0682 | 1.3 | 0.6;3.0 | 0.552 |
|  | **Adults** | 2.5 | 0.9;7.9 | 0.098 | 1.5 | 0.4 ;5.0 | 0.521 |
|  | **Children** | 0.5 | 0.1;2.7 | 0.427 |  |  |  |
| **Solid tumor** | **All** | 1.2 | 0.4;3.1 | 0.740 | 1.2 | 0.4;3.4 | 0.677 |
|  | **Adults** | 1.5 | 0.4;5.0 | 0.526 | 1.0 | 0.3;3.7 | 0.989 |
|  | **Children** | 0.7 | 0.1;4.4 | 0.672 | - | - | - |
| **Chronic immunosuppressive medication** | **All** | 4.2 | 1.5;13.0 | 0.009 | 3.3 | 1.1;10.6 | 0.033 |
|  | **Adults** | 5.2 | 1.6;20.4 | 0.010 | 3.0 | 0.8;13.0 | 0.121 |
|  | **Children** | 2.0 | 0.2;21.4 | 0.535 | - | - | - |
| **PID** | **All** | 0.7 | 0.1;3.9 | 0.689 | 0.9 | 0.1;5.5 | 0.894 |
|  | **Children** | 0.7 | 0.1;6.4 | 0.725 | - | - | - |

| **Outcome: Pneumonia**  **N= 203 (152 adults, 51 children)*** | | **Univariate analyses** | | | **Multivariate analyses** | | |
| --- | --- | --- | --- | --- | --- | --- | --- |
|  |  | **OR** | **95 %C.I** | **P value** | **OR** | **95 % C.I** | **P value** |
| **Increasing age (by 10Y range)** | **All** | 1.2 | 1.1;1.4 | <0.001 | 1.2 | 1.1;1.4 | 0.002 |
| **Bacterial co-infection** | **All** | 3.3 | 1.6;6.9 | 0.002 | 3.7 | 1.6;8.7 | 0.002 |
| **nALC** | **All** | 1.0 | 1.0;1.1 | 0.272 | 1.0 | 1.0;1.2 | 0.450 |
| **Type immunosuppression (compared to HSCT)** | | | | | | | |
| **SOT** | **All** | 0.8 | 0.3;1.9 | 0.623 | 0.5 | 0.2;1.3 | 0.148 |
| **Leukaemia/lymphoma** | **All** | 1.0 | 0.4;2.3 | 0.970 | 1.2 | 0.5;2.9 | 0.760 |
| **Solid tumor** | **All** | 1.3 | 0.5;3.6 | 0.567 | 1.4 | 0.5;4.2 | 0.517 |
| **Chronic immunosuppressive medication** | **All** | 2.2 | 0.8;6.1 | 0.109 | 1.5 | 0.5;4.3 | 0.461 |
| **PID** | **All** | 0.4 | 0.0;3.1 | 0.478 | 0.8 | 0.0;6.0 | 0.825 |

**Legend:** Absolute lymphocyte counts (ALC); Hematopoietic stem cell recipients (HSCT), Primary immunodeficiency disease (PID) ; Solid organ transplant (SOT); N refers to the total number of patients included in all multivariable analyses for whom information on all predictors and outcomes was available.

**Addendum Tables S4 and S5 refer to Post-hoc sensitivity analyses excluding patients with viral co-infection.**

**Addendum Table S4**

**Predictors for ARTI-attributable hospital admission among all immunocompromises patients and stratified by age-groups (adults vs. children)**

| **N=166 (121 adults, 45 children)*** | | **Univariate analyses** | | | **Multivariate analyses** | | |
| --- | --- | --- | --- | --- | --- | --- | --- |
|  |  | **OR** | **95 %C.I** | **P value** | **OR** | **95 % C.I** | **P value** |
| **Increasing age (by 10Y range)** | **All** | 1.1 | 0.9 ;1.2 | 0.267 | 1.1 | 0.9;1.2 | 0.277 |
|  | **Adults** | 1.7 | 1.3;2.4 | <0.001 | 1.4 | 1.1;2.1 | 0.030 |
|  | **Children** | 0.5 | 0.1;1.7 | 0.300 | - | - | - |
| **Bacterial co-infection** | **All** | 1.9 | 0.8;4.1 | 0.121 | 1.7 | 0.7;4.0 | 0.263 |
|  | **Adults** | 2.1 | 0.9;5.3 | 0.102 | 1.6 | 0.6;4.8 | 0.374 |
|  | **Children** | 1.3 | 0.3;6.3 | 0.728 | 1.9 | 0.3;11.6 | 0.470 |
| **nALC** | **All** | 1.0 | 1.0;1.2 | 0.403 | 1.0 | 1.0;NA | 0.508 |
|  | **Adults** | 1.0 | 1.0;NA | 0.407 | 1.0 | 1.0;1.3 | 0.533 |
|  | **Children** | 0.8 | 0.5;1.3 | 0.367 | 0.7 | 0.4;1.2 | 0.273 |
| **Type immunosuppression (Reference: HSCT)** | | | | | | | |
| **SOT** | **All** | 3.2 | 1.1;11.0 | 0.044 | 2.8 | 0.9;9.9 | 0.082 |
|  | **Adults** | 3.2 | 1.1;11.0 | 0.052 | 2.7 | 0.8;9.9 | 0.114 |
| **Leukemia/lymphoma** | **All** | 5.9 | 2.0;19.9 | 0.002 | 6.4 | 2.1;22.6 | 0.002 |
|  | **Adults** | 4.0 | 1.0;16.6 | 0.051 | 2.1 | 0.4;9.8 | 0.344 |
| **Solid tumor** | **All** | 9.9 | 2.8;39.7 | <0.001 | 10.2 | 2.9;41.4 | <0.001 |
|  | **Adults** | 14.8 | 3.5;75.6 | <0.001 | 11.5 | 2.6;60.5 | 0.002 |
| **Chronic immunosuppressive medication** | **All** | 16.8 | 4.7;70.8 | <0.001 | 14.0 | 3.8;60.2 | <0.001 |
|  | **Adults** | 14.5 | 3.6;66.4 | <0.001 | 6.2 | 1.3;32.7 | 0.023 |
| **PID** | **All** | 7.2 | 0.7;72.7 | 0.075 | 8.4 | 0.8;90.8 | 0.064 |

**Legend:** Absolute lymphocyte counts (ALC), Hematopoietic stem cell recipients (HSCT), Primary immunodeficiency disease (PID) Solid organ transplant (SOT), acute-respiratory-tract infection (ARTI); N refers to the total number of patients included in all multivariable analyses for whom information on all predictors and outcomes was available

**Addendum Table S5**

**Predictors of progression to LRTI and pneumonia among all immunocompromises patients and stratified by age-groups (adults vs. children).**

| **Outcome: LRTI**  **N= 166 (121 adults, 45 children)*** | | **Univariate analyses** | | | **Multivariate analyses** | | |
| --- | --- | --- | --- | --- | --- | --- | --- |
|  |  | **OR** | **95 %C.I** | **P value** | **OR** | **95 % C.I** | **P value** |
| **Increasing age**  **(by 10Y range)** | **All** | 1.1 | 1.0;1.3 | 0.043 | 1.1 | 1.0;1.3 | 0.156 |
|  | **Adults** | 1.4 | 1.1;1.8 | 0.004 | 1.3 | 1.0;1.7 | 0.102 |
|  | **Children** | 0.7 | 0.2;2.3 | 0.539 | - | - | - |
| **Bacterial co-infection** | **All** | 2.6 | 1.1;6.0 | 0.025 | 2.9 | 1.2;7.3 | 0.021 |
|  | **Adults** | 2.7 | 1.1;7.5 | 0.045 | 3.0 | 1.1;9.1 | 0.045 |
|  | **Children** | 2.2 | 0.5;12.0 | 0.329 | 2.5 | 0.5;17.0 | 0.287 |
| **nALC** | **All** | 1.0 | 1.0;1.3 | 0.535 | 1.1 | 1.0;1.4 | 0.602 |
|  | **Adults** | 1.0 | 1.0;1.3 | 0.579 | 1.1 | 1.0;1.4 | 0.681 |
|  | **Children** | 1.0 | 0.6;1.6 | 0.950 | 0.9 | 0.5;1.5 | 0.644 |
| **Type immunosuppression (compared to HSCT)** | | | | | | | |
| **SOT** | **All** | 0.9 | 0.4;2.1 | 0.750 | 0.7 | 0.3;1.6 | 0.370 |
|  | **Adults** | 0.9 | 0.4;2.3 | 0.896 | 0.7 | 0.2;1.8 | 0.451 |
|  | **Children** | 0.3 | 0.0 ;6.1 | 0.472 | - | - | - |
| **Leukemia/lymphoma** | **All** | 1.2 | 0.5;2.8 | 0.742 | 1.3 | 0.5;3.4 | 0.590 |
|  | **Adults** | 2.7 | 0.8;10.1 | 0.113 | 1.8 | 0.5;7.0 | 0.386 |
|  | **Children** | 0.3 | 0.0;3.6 | 0.353 | - | - | - |
| **Solid tumor** | **All** | 1.6 | 0.5;4.9 | 0.407 | 1.6 | 0.5;5.2 | 0.396 |
|  | **Adults** | 1.4 | 0.4;5.3 | 0.571 | 1.1 | 0.3;4.1 | 0.943 |
|  | **Children** | 1.0 | 0.0;19.3 | 1.000 | - | - | - |
| **Chronic immunosuppressive medication** | **All** | 3.5 | 1.1;12.3 | 0.039 | 2.6 | 0.8;9.5 | 0.124 |
|  | **Adults** | 3.7 | 1.1;15.2 | 0.048 | 1.9 | 0.5;9.1 | 0.390 |
|  | **Children** | 1.5 | 0.0;57.1 | 0.810 | - | - | - |
| **PID** | **All** | 0.4 | 0.0;3.3 | 0.426 | 0.4 | 0.0;3.8 | 0.461 |
|  | **Children** | 0.3 | 0.0;6.6 | 0.423 | - | - | - |

| **Outcome: Pneumonia**  **N= 166 (121 adults, 45 children)*** | | **Univariate analyses** | | | **Multivariate analyses** | | |
| --- | --- | --- | --- | --- | --- | --- | --- |
|  |  | **OR** | **95 %C.I** | **P value** | **OR** | **95 % C.I** | **P value** |
| **Increasing age (by 10Y range)** | **All** | 1.2 | 1.1;1.4 | 0.002 | 1.2 | 1.1;1.4 | 0.009 |
| **Bacterial co-infection** | **All** | 2.8 | 1.3;6.1 | 0.012 | 3.5 | 1.4;9.0 | 0.006 |
| **nALC** | **All** | 1.0 | 1.0;1.1 | 0.370 | - | - | - |
| **Type immunosuppression (compared to HSCT)** | | | | | | | |
| **SOT** | **All** | 0.8 | 0.3;2.0 | 0.580 | 0.5 | 0.2;1.4 | 0.208 |
| **Leukemia/lymphoma** | **All** | 1.0 | 0.4;2.7 | 0.939 | 1.3 | 0.5;3.5 | 0.656 |
| **Solid tumor** | **All** | 2.5 | 0.7;9.5 | 0.158 | 2.0 | 0.5;7.8 | 0.310 |
| **Chronic immunosuppressive medication** | **All** | 2.2 | 0.7;6.6 | 0.170 | 1.3 | 0.4;4.2 | 0.660 |
| **PID** | **All** | - | - | - | - | - | - |

**Legend:** Absolute lymphocyte counts (ALC); Hematopoietic stem cell recipients (HSCT), Primary immunodeficiency disease (PID) ; Solid organ transplant (SOT); N refers to the total number of patients included in all multivariable analyses for whom information on all predictors and outcomes was available.

**Addendum Tables S6 and S7 refer to *Post-hoc* sensitivity analyses excluding children detected by rapid antigen assays.**

**Addendum table S6**

**Predictors for ARTI-attributable hospital admission among all immunocompromised patients and stratified by age-groups (adults vs. children)**

| **N=203 (152 adults, 51 children)*** | | **Univariate analyses** | | | **Multivariate analyses** | | |
| --- | --- | --- | --- | --- | --- | --- | --- |
|  |  | **OR** | **95 %C.I** | **P value** | **OR** | **95 % C.I** | **P value** |
| **Increasing age (by 10Y range)** | **All** | 1.1 | 1.0;1.2 | 0.162 | 1.1 | 0.9;1.2 | 0.252 |
|  | **Adults** | 1.6 | 1.3;2.1 | <0.001 | 1.4 | 1.0;1.8 | 0.027 |
|  | **Children** | 0.4 | 0.1;1.5 | 0.207 | - | - | - |
| **Bacterial co-infection** | **All** | 1.8 | 0.9;3.8 | 0.115 | 1.6 | 0.7;3.6 | 0.284 |
|  | **Adults** | 2.2 | 0.9;5.2 | 0.066 | 1.7 | 0.6;4.5 | 0.286 |
|  | **Children** | 1.0 | 0.2;5.0 | 0.987 | 1.5 | 0.2;9.2 | 0.677 |
| **nALC** | **All** | 1.0 | 1.0;1.2 | 0.306 | 1.0 | 1.0;1.2 | 0.402 |
|  | **Adults** | 1.0 | 1.0;1.2 | 0.304 | 1.0 | 1.0;1.2 | 0.436 |
|  | **Children** | 0.8 | 0.5;1.2 | 0.228 | 0.7 | 0.4;1.1 | 0.201 |
| **Type immunosuppression (Reference: HSCT)** | | | | | | | |
| **SOT** | **All** | 2.2 | 0.9;5.6 | 0.072 | 1.9 | 0.8;4.9 | 0.153 |
|  | **Adults** | 2.1 | 0.9;5.4 | 0.105 | 1.6 | 0.6;4.4 | 0.312 |
| **Leukaemia/lymphoma** | **All** | 3.3 | 1.4;8.3 | 0.010 | 3.2 | 1.3;8.4 | 0.015 |
|  | **Adults** | 3.0 | 1.0;9.3 | 0.057 | 1.5 | 0.4;5.2 | 0.556 |
| **Solid tumor** | **All** | 8.1 | 2.8;25.2 | <0.001 | 8.4 | 2.9;26.4 | <0.001 |
|  | **Adults** | 7.4 | 2.1;28.4 | 0.002 | 5.2 | 1.4;20.9 | 0.015 |
| **Chronic immunosuppressive medication** | **All** | 10.6 | 3.7;33.8 | <0.001 | 9.2 | 3.1;29.9 | <0.001 |
|  | **Adults** | 8.2 | 2.6;28.4 | <0.001 | 4.1 | 1.1;16.0 | 0.034 |
| **PID** | **All** | 4.6 | 0.8;28.2 | 0.082 | 5.6 | 0.9;36.3 | 0.063 |

**Legend:** Absolute lymphocyte counts (ALC), Hematopoietic stem cell recipients (HSCT), Primary immunodeficiency disease (PID) Solid organ transplant (SOT), acute-respiratory-tract infection (ARTI)**;** N refers to the total number of patients included in all multivariable analyses for whom information on all predictors and outcomes was available.

**Addendum Table S7**

**Predictors of progression to LRTI and pneumonia among all immunocompromised patients and stratified by age-groups (adults vs. children).**

| **Outcome: LRTI**  **N= 203 (152 adults, 51 children)*** | | **Univariate analyses** | | | **Multivariate analyses** | | |
| --- | --- | --- | --- | --- | --- | --- | --- |
|  |  | **OR** | **95 %C.I** | **P value** | **OR** | **95 % C.I** | **P value** |
| **Increasing age**  **(by 10Y range)** | **All** | 1.2 | 1.1;1.4 | 0.001 | 1.2 | 1.0;1.3 | 0.014 |
|  | **Adults** | 1.3 | 1.1;1.7 | 0.006 | 1.2 | 1.0;1.5 | 0.128 |
|  | **Children** | 0.8 | 0.2;2.7 | 0.724 | - | - | - |
| **Bacterial co-infection** | **All** | 3.2 | 1.5;7.3 | 0.005 | 3.2 | 1.4;7.9 | 0.008 |
|  | **Adults** | 3.2 | 1.3;8.6 | 0.014 | 3.4 | 1.3;9.8 | 0.018 |
|  | **Children** | 2.9 | 0.6;16.2 | 0.206 | 3.7 | 0.6;26.2 | 0.155 |
| **nALC** | **All** | 1.0 | 1.0;1.2 | 0.365 | 1.0 | 1.0;1.2 | 0.502 |
|  | **Adults** | 1.1 | 1.0;1.3 | 0.403 | 1.0 | 1.0;1.3 | 0.580 |
|  | **Children** | 0.9 | 0.6;1.4 | 0.796 | 0.8 | 0.5;1.3 | 0.461 |
| **Type immunosuppression (compared to HSCT)** | | | | | | | |
| **SOT** | **All** | 1.0 | 0.5;2.1 | 0.978 | 0.7 | 0.3;1.6 | 0.378 |
|  | **Adults** | 1.0 | 0.4;2.1 | 0.923 | 0.7 | 0.3;1.5 | 0.344 |
|  | **Children** | 1.3 | 0.1;17.6 | 0.819 | - | - | - |
| **Leukaemia/lymphoma** | **All** | 1.0 | 0.4;2.1 | 0.900 | 1.0 | 0.4;2.3 | 0.947 |
|  | **Adults** | 2.0 | 0.7;6.0 | 0.211 | 1.2 | 0.4;3.9 | 0.791 |
|  | **Children** | 0.8 | 0.1;6.4 | 0.771 | - | - | - |
| **Solid tumor** | **All** | 1.0 | 0.4;2.7 | 0.981 | 1.0 | 0.4;2.8 | 0.989 |
|  | **Adults** | 1.2 | 0.4;3.8 | 0.811 | 0.8 | 0.2;2.8 | 0.718 |
|  | **Children** | 1.2 | 0.1;12.8 | 0.872 | - | - | - |
| **Chronic immunosuppressive medication** | **All** | 3.4 | 1.2;10.6 | 0.022 | 2.7 | 0.9;8.5 | 0.081 |
|  | **Adults** | 4.0 | 1.3;15.6 | 0.026 | 2.4 | 0.7;10.2 | 0.204 |
|  | **Children** | 3.0 | 0.3;44.0 | 0.383 | - | - | - |
| **PID** | **All** | 0.6 | 0.1;3.4 | 0.580 | 0.9 | 0.1;5.4 | 0.896 |
|  | **Children** | 1.3 | 0.1;17.6 | 0.819 | - | - | - |

| **Outcome: Pneumonia**  **N= 203 (152 adults, 51 children)*** | | **Univariate analyses** | | | **Multivariate analyses** | | |
| --- | --- | --- | --- | --- | --- | --- | --- |
|  |  | **OR** | **95 %C.I** | **P value** | **OR** | **95 % C.I** | **P value** |
| **Increasing age (by 10Y range)** | **All** | 1.2 | 1.1;1.4 | <0.001 | 1.2 | 1.1;1.4 | 0.004 |
| **Bacterial co-infection** | **All** | 2.7 | 1.3;5.7 | 0.010 | 3.0 | 1.3;7.0 | 0.011 |
| **nALC** | **All** | 1.0 | 1.0;1.1 | 0.278 | 1.0 | 1.0;1.1 | 0.449 |
| **Type immunosuppression (compared to HSCT)** | | | | | | | |
| **SOT** | **All** | 0.8 | 0.3;1.7 | 0.513 | 0.5 | 0.2;1.2 | 0.138 |
| **Leukaemia/lymphoma** | **All** | 1.1 | 0.5;2.5 | 0.842 | 1.1 | 0.4;2.8 | 0.821 |
| **Solid tumor** | **All** | 1.5 | 0.5;4.0 | 0.465 | 1.5 | 0.5;4.2 | 0.488 |
| **Chronic immunosuppressive medication** | **All** | 1.9 | 0.7;5.2 | 0.189 | 1.3 | 0.4;3.7 | 0.627 |
| **PID** | **All** | 0.4 | 0.0;2.8 | 0.442 | 0.7 | 0.0;5.5 | 0.779 |

**Legend:** Absolute lymphocyte counts (ALC); Hematopoietic stem cell recipients (HSCT), Primary immunodeficiency disease (PID) ; Solid organ transplant (SOT); N refers to the total number of patients included in all multivariable analyses for whom information on all predictors and outcomes was available.
